# Draft Genome Sequence of *Mizugakiibacter sediminis* skMP5^T^

**DOI:** 10.1128/genomeA.01185-15

**Published:** 2015-10-08

**Authors:** Tomohiro Watanabe, Hisaya Kojima, Manabu Fukui

**Affiliations:** The Institute of Low Temperature Science, Hokkaido University, Sapporo, Japan

## Abstract

Strain skMP5^T^ is a moderately thermophilic and facultatively anaerobic bacterium, described as a representative of *Mizugakiibacter sediminis*. Here, we report the annotated draft genome sequence of strain skMP5^T^.

## GENOME ANNOUNCEMENT

*Mizugakiibacter sediminis* is a heterotrophic bacterium belonging to the family *Xanthomonadaceae* within the class *Gammaproteobacteria* (1). The type strain is skMP5^T^, which was isolated from sediment of a freshwater lake in Japan. The 16S rRNA gene sequence of strain skMP5^T^ shares only 93% identity with its closest cultured relative, *Tahibacter aquaticus* RaM5-2. Strain skMP5^T^ is moderately thermophilic and has a wide temperature range for growth from 25°C to 52°C (optimum 48°C to 50°C). The strain is facultatively anaerobic and can reduce nitrate to nitrite under anoxic conditions. Here, the annotated genome of strain skMP5^T^ is reported.

Genomic DNA was extracted using the Wizard genomic DNA purification kit (Promega). For next-generation sequencing, a DNA library with 350-bp inserts was prepared using the TruSeq Nano DNA library prep kit (Illumina). The library was sequenced using paired-end 100-bp reads on an Illumina Hiseq. A total of 10,581,839 high-quality filtered reads were assembled *de novo* using Velvet version 1.2.08, with a hash length of 45 bp. The resulting assembly comprised 149 scaffolds (containing 179 contigs) totaling 3.1 Mb in size. The calculated G+C content of the draft genome was 71%. The genome was annotated automatically using the Microbial Gnome Annotation Pipeline (2). As a result, a total of 2,837 genes, 45 tRNAs, and 1 rRNA operon were predicted.

In the original description of *Mizugakiibacter sediminis* skMP5^T^, its growth substrate could not be determined because growth of the strain occurred only in the presence of tryptone. The annotated genome will advance our understanding of its physiological characteristics, such as carbon source utilization.

### Nucleotide sequence accession numbers.

The draft genome sequence of *Mizugakiibacter sediminis* skMP5^T^ has been deposited in DDBJ/EMBL/GenBank under the accession number BBUM00000000. The version described in this paper is the second version, BBUM02000000.
